# Erratum for “Retraction statement: Prevalence of sexually transmitted infections and associated ‎risk behaviors in prisoners: A systematic review”

**DOI:** 10.1002/hsr2.1851

**Published:** 2024-01-24

The following retraction, published online on {First published: 25 July 2023} in Wiley Online Library {https://onlinelibrary.wiley.com/doi/10.1002/hsr2.1458}. The retraction notice has been amended, and the online version has been revised to reflect the correction.

We apologize for this error.

